# Effects of hydrazone-based G-quadruplex ligands on *FANCJ/BRIP1*-depleted cancer cells and a *Caenorhabditis elegans dog-1^−/−^* strain

**DOI:** 10.1093/narcan/zcaf004

**Published:** 2025-02-08

**Authors:** Marcello Germoglio, Federica D’Aria, Giuseppe Cortone, Antonello Prodomo, Mohammad Mahtab, Rita Morigi, Jussara Amato, Francesca M Pisani, Concetta Giancola

**Affiliations:** Department of Pharmacy, University of Naples Federico II, Naples 80131, Italy; Istituto di Biochimica e Biologia Cellulare, Consiglio Nazionale delle Ricerche, Naples 80131, Italy; Department of Pharmacy, University of Naples Federico II, Naples 80131, Italy; Istituto di Biochimica e Biologia Cellulare, Consiglio Nazionale delle Ricerche, Naples 80131, Italy; Istituto di Biochimica e Biologia Cellulare, Consiglio Nazionale delle Ricerche, Naples 80131, Italy; Istituto di Biochimica e Biologia Cellulare, Consiglio Nazionale delle Ricerche, Naples 80131, Italy; Department of Pharmacy and Biotechnology, Alma Mater Studiorum - University of Bologna, Bologna 40126, Italy; Department of Pharmacy, University of Naples Federico II, Naples 80131, Italy; Istituto di Biochimica e Biologia Cellulare, Consiglio Nazionale delle Ricerche, Naples 80131, Italy; Department of Pharmacy, University of Naples Federico II, Naples 80131, Italy

## Abstract

G-quadruplex (G4) DNAs are alternative nucleic acid structures, proposed to play important roles in regulating DNA replication, gene transcription, and translation. Several specialized DNA helicases are involved in cellular G4 metabolism, in some cases with redundant functions. Among them, human FANCJ/BRIP1, which has orthologs in all metazoans, is one of the most powerful G4 resolvases, believed to act mainly at DNA replication forks. Here, we tested the effects of a set of hydrazone-derivative G4 ligands in a *FANCJ*-knocked-out HeLa cell line and in a *Caenorhabditis elegans* strain, where DOG-1, a FANCJ ortholog, was disrupted, as a whole organism model system. Our results revealed that loss of FANCJ specifically sensitized cancer cells to FIM-15, a mono-guanylhydrazone derivative bearing the diimidazopyrimidine core, among the tested hydrazone-based compounds and induced enhanced DNA damage in different chromosomal sites including telomeric ends. Moreover, dietary administration of FIM-15 to *dog-1*^−/−^ nematodes stabilized G4 structures in gonadal cell nuclei and resulted in compromised embryonic development in the first-generation post-treatment. Collectively, our findings unveil a specific vulnerability of *FANCJ*-knocked-out cancer cells (and DOG-1-lacking worms) to G4 stabilization by the FIM-15 compound. This study provides an important proof-of-principle for use of G4 ligands in synthetic lethality-based therapeutic approaches targeting FANCJ-defective cancer cells.

## Graphical Abstract

**Graphical Abstract:**
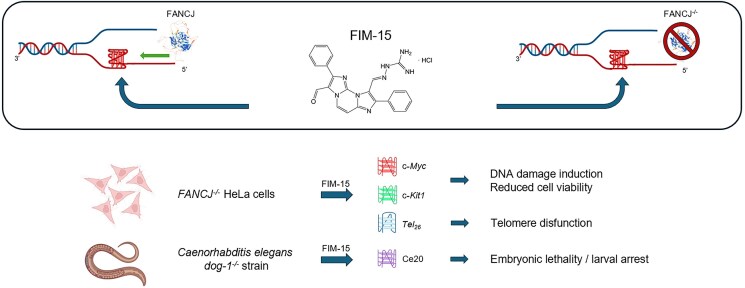

## Introduction

Guanine-rich (G-rich) sequences of DNA fold into non-Watson–Crick structures called G-quadruplexes (G4s). These structures are formed by stacking of guanine quartets that are stabilized by Hoogsteen hydrogen bonds and monovalent cations, preferentially Na^+^ and K^+^ [1, 2]. G4s are important conformational ‘knots’ that are located near gene transcriptional start sites, at telomeres and DNA replication origins, where they play a role in the initiation step of DNA replication and cell proliferation [3]. They were extensively studied by nuclear magnetic resonance [4, 5], X-ray crystallography [6], and other bio-physicochemical methodologies [7, 8], in terms of structural topologies and dynamics. G4s were reported within the promoter of several genes, like insulin [9], *MYC* [10, 11], *VEGF* [12], *HIF*-1α [13], *RET* [14], *BCL*-2 [15], *KIT* [16, 17], and *KRAS* [18]. It was shown that G4 formation is higher in proto-oncogenes than in tumour-suppressor genes. Notably, the G-rich single-stranded overhang of human telomeres is highly prone to form G4s with different folding topologies. Therefore, G4s represent important targets for developing anti-cancer drugs that interfere with different cell informational processes by binding and stabilizing these alternative DNA structures, so impeding their resolution [3]. The stabilizing effect of an ever-growing number of G4-binding small molecules (usually hydrophobic, aromatic, and planar molecules) can be exploited for selective gene suppression. Recently, Capranico *et al.* found that pyridostatin (PDS) [19] and PhenDC3 [20], two well-known G4 binders, are also cytostatic modulators of innate immune genes in cancer cells [21]. In addition, some of us developed hydrazone-based G4 binders and demonstrated that their cytotoxic effect is correlated with their ability to bind and stabilize G4s both *in vitro* and in cells [22–24].

G4 formation and unwinding are critical processes within the cell, and their dysregulation has a major impact in genome stability and cancer onset. In particular, several nucleic acid helicases have been shown to target and regulate G4 structures, and hence play a key role in G4 metabolism [3].

G4 ligands can impact helicase activity through G4 stabilization [25–27]. However, the interplay among G4, ligands, and helicases is still partially unexplored. Its thorough *in vitro* and *in vivo* characterization could improve the understanding of reciprocal influence each part has in the helicase-driven modulation of the G4s biological functions.

Studies carried out in different systems (*Caenorhabditis elegans* [28], chicken DT40 [29] and human cells [30], and *Xenopus laevis* cell-free egg extracts [31, 32]) revealed that the FANCJ DNA helicase (DOG-1 in nematodes) has an evolutionarily conserved prominent role in resolving G4 DNA structures and other DNA replication roadblocks, such as interstrand DNA cross-links and protein–DNA adducts, which generate replication stress, if not timely repaired [33]. *dog-1* mutant worms showed germline as well as somatic deletions in genes containing poly-guanine tracts, a phenotype leading to the proposal that the DOG-1 protein resolves the DNA secondary structures arising at G-rich genomic *loci* [28]. Human FANCJ, also known as BRIP1 (for BRCA1-interacting protein 1), belongs to the group of super-family 2 (SF2) iron–sulfur (Fe–S) cluster-containing DNA helicases. *FANCJ*/*BRIP1* is frequently mutated in breast and ovarian cancers, as well as in other tumour types [34–36]. Moreover, bi-allelic mutations of the *FANCJ* gene cause Fanconi anaemia, a rare hereditary disease, characterized by hematopoietic stem cell defects, progressive bone marrow failure, genomic instability, and cancer predisposition [37–40]. The purified human FANCJ recombinant protein is able to dismantle G4 DNA structures having different topology with a 5′–3′ directionality in an ATPase-dependent manner *in vitro* [33]. Moreover, it was shown that *FANCJ*-knocked-out cells treated with telomestatin, a well-known G4 ligand, exhibited reduced proliferation, apoptosis, and enhanced DNA damage [41].

**Figure 1.**
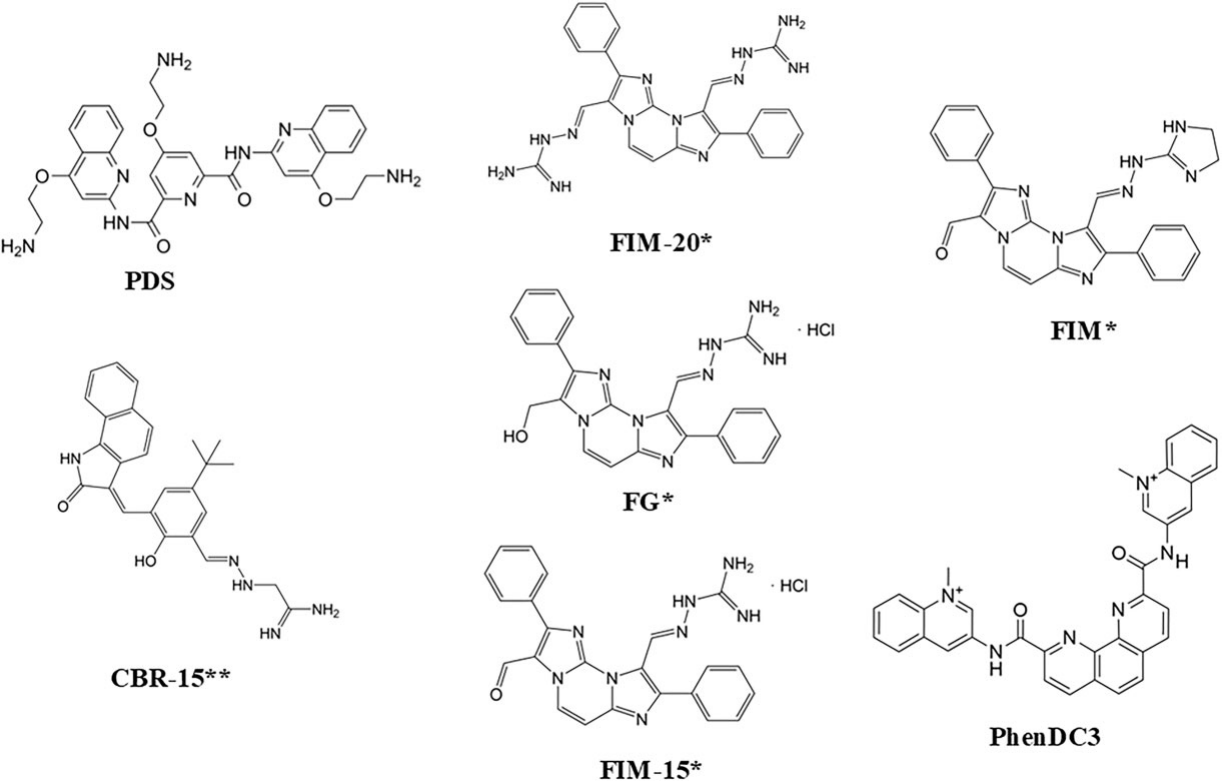
Chemical structures of the G4 ligands used in this study. The compounds labelled with * or ** were previously described in [22, 24], respectively.

In this study, we investigated the role of FANCJ in G4 DNA metabolism either *in vitro*, in HeLa cell lines, or *in vivo*, using *C. elegans* as a whole organism model. We examined different G4 stabilizers (Fig. 1), including the well-known G4 binders PDS and PhenDC3, along with a selection of hydrazone derivatives bearing a benzoindolinone or a diimidazopyrimidine nucleus and one or, in the case of FG, two nitrogen chains, which can be a hydrazinoimidazoline for FIM or an iminoguanidine for the guanylhydrazones CBR-15, FG, FIM-15, and FIM-20 [22, 24]. The G4 ligands were tested in a *FANCJ*-knocked-out HeLa cell line or in a *dog-1^−/-^ C. elegans* strain (*dog-1* is the nematode ortholog of human *FANCJ*) [28, 42], to study their effects on viability, DNA damage induction, and telomere maintenance. We found that, among the different G4 ligands tested, FIM-15 was the only one that specifically reduced relative viability, enhanced DNA damage, and impaired telomere integrity of *FANCJ*-KO cells. Moreover, FIM-15 increased G4 *foci* in gonadal germ cells and adversely impacted embryonic development, when dietary administered to *dog-1^−/−^* nematodes.

Overall, our study provides new insights into the role of the FANCJ DNA helicase in cellular G4 DNA metabolism and offers a glimpse of the anti-cancer potential of G4 stabilizers in *FANCJ*-defective tumours.

## Materials and methods

### G4 stabilizers

PDS and PhenDC3 are commercially available compounds (Merck Life Science). The hydrazone derivatives FIM, FG, CBR-15, FIM-15, and FIM-20 were synthetized at the Department of Pharmacy and Biotechnology of the University of Bologna. The molecular structures of the G4 ligands are reported in Fig. 1.

### Cell lines

HeLa cell lines were cultured in Dulbecco’s Modified Eagle’s Medium high glucose supplemented with 10% fetal bovine serum and antibiotics. *FANCJ*- and *DDX11*-KO cells were previously described [43, 44].

### Expression and purification of BG4 single-chain antibody

Recombinant 6xHis/3xFLAG-tagged BG4 was produced in *Escherichia coli* using pSANG10-3F-BG4 (Addgene; #55756) as follows: BL21(DE3) competent cells were transformed with pSANG10-3F plasmid expressing BG4 scFv anti-G4 antibody. The transformed cells were grown in 3 L of 2× TY medium [1.6% (w:v) bacto tryptone, 1% (w:v) bacto yeast extract, 0.5% (w:v) NaCl] containing 1% (w:v) glucose and 50 μg/mL kanamycin, for 24 h at 18°C. Bacterial cells were centrifugated for 30 min at 4000 × *g* (4°C). Cell pellet was lysed in 300 mL of TES buffer [50 mM Tris–HCl, 1 mM ethylenediaminetetraacetic acid (EDTA), 20% (w:v) sucrose] on ice and stirred in presence of EDTA-free protease inhibitor cocktail (cOmplete™ Protease Inhibitor Cocktail; Roche) for 15 min at 4°C. The cell extract was diluted 1:1 with 1:5 TES buffer [10 mM Tris–HCl, 0.2 mM EDTA, 4% (w:v) sucrose] containing 2 mM MgSO_4_ and benzonase (2.5 units/mL) and gently stirred for 30 min at 4°C, prior to centrifugation for 20  min at 8000 × *g* (4°C). The supernatant was filtered (0.45 μm) and the antibody was purified using HisPur™ Cobalt Superflow Agarose resin (Thermo Fisher Scientific) pre-equilibrated in phosphate-buffered saline (PBS) containing 20 mM imidazole. Supernatant and 3 mL of resin were mixed in a glass baker with magnetic bar for 30 min at 4°C, then transferred into a chromatography column. The resin was washed twice with 3-column volume PBS supplemented with 100  mM NaCl and 10  mM imidazole. The BG4 antibody was eluted with PBS 1× supplemented with 250  mM imidazole (pH 8.0). The elution buffer was exchanged with inner cell salt buffer [25  mM HEPES–NaOH (pH 7.6), 110  mM KCl, 10.5  mM NaCl, 1  mM MgCl_2_]. The eluted fractions containing the BG4 antibody were pooled, and the sample was concentrated using an Amicon Ultra-15 Centrifugal Filter Unit with 10-kDa cutoff (Millipore). Protein concentration and quality was checked by sodium dodecyl sulfate–polyacrylamide gel electrophoresis.

### Immunofluorescence

To analyse BG4 *foci*, HeLa cells were grown for 24 h on coverslips in six-well plates (3 × 10^5^ cells/well) in absence or presence of G4 stabilizers as indicated in the figure legends. Coverslips were fixed in cold methanol for 8  min on ice and further blocked for 1  h at room temperature (1 h/RT) with PBS containing 1% (w:v) bovine serum albumin (BSA), 0.5% (w:v) fish gelatine, and 0.1% (v:v) Tween-20. Thereafter, coverslips were incubated overnight with BG4 single-chain antibody (1 ng/μL) in a humid chamber at 4°C. After washing with 1× PBS, sensitive detection was achieved through an amplified fluorescence signal generated by incubation for 1 h/RT with anti-FLAG antibody produced in rabbit (Sigma–Aldrich; Catalogue No. F7425) at 5 ng/μL, thereafter, anti-rabbit Alexa Fluor™ 488 (5 ng/μL; Thermo Fisher Scientific) as a tertiary fluorochrome-labelled antibody (1 h/RT). Coverslips were mounted into glass slides using mounting media containing DAPI (5 ng/μL). Images were acquired with a confocal microscope (Zeiss LSM 980) using a ×63 magnification objective (Nikon). BG4 *foci* were quantified with Fiji software using the following formula to calculate the corrected total fluorescence (CTF): CTF = integrated density – (area of selected nuclei × mean fluorescence of background readings).

### XTT viability assay

2,3-bis-(2-methoxy-4-nitro-5-sulfophenyl)-2H-tetrazolium-5-carboxanilide (XTT; Invitrogen, X6493) assay was performed to determine cell viability after treating cell lines with G4 stabilizers (PDS, FG, FIM, CBR-15, FIM-15, and FIM-20; Fig. 1). For this, 4 × 10^3^ control or 5 × 10^3^*FANCJ*-KO HeLa cells were seeded in 96-well plates. After 4 h, different increasing concentrations of G4 stabilizers were added to three wells containing control or *FANCJ*-KO HeLa cells. Cell cultures were grown for 72 h. Thereafter, the XTT solution (50 μL) was added to each well. XTT is metabolically reduced in viable cells to an orange formazan product, as previously described [45]. Thus, after a 3-h incubation, absorbance was measured at a wavelength of 450 nm and non-specific absorbance is measured at a wavelength of 630 nm using the Victor Nivo™ multimode plate reader (Perkin Elmer). Cell viability was calculated as follows: cell viability = (A_450nm_ sample – A_630nm_ sample) – (A_450nm_ blank – A_630nm_ blank). Then, the cell viability in the absence of G4 stabilizer was used as a reference to calculate relative cell viability and IC_50_ was determined using a non-linear regression model with GraphPad Prism 9.0 (Supplementary Table S1).

### Fluorescence *in situ* hybridization analysis

HeLa cells were grown for 24 h on coverslips in six-well plates (3 × 10^5^ cells/well). Coverslips were incubated in cytoplasmic extraction buffer [20  mM HEPES–KOH (pH 7.9), 20  mM NaCl, 5  mM MgCl_2_, 30  mM sucrose, 0.5% (v:v) NP-40] for 10 min. Slides were washed very gently once with 1× PBS containing 0.1% (v:v) Tween-20, once with 1× PBS, and then fixed in cold Carnoy fixative [3:1 (v:v) methanol/acetic acid] for 8 min on ice. Slides were washed three times in 1× PBS, then incubated in pre-warmed pepsin solution (1 mg/mL in 1× PBS) for 5 min at 37°C. Thereafter, a fixation step was carried out with a solution containing 2% (v:v) paraformaldehyde for 2 min at RT. After three washing steps in 1× PBS, coverslips were treated for 90 min at 37°C with RNase A (at 50 μg/mL in 1× PBS supplemented with 0.1 M glycine). Coverslips were washed in 1× PBS, then dehydrated with 70%, 90%, and 100% ethanol (2  min each), followed by air-drying. Thereafter, slides were overlaid with 50  nM of TelG-Cy3 peptide nucleic acid (PNA; Cy3-OO-KKK-ttagggttagggtt) in hybridization buffer [10 mM Tris–HCl, 70% (v:v) formamide, 1× Roche blocking reagent diluted in 100 mM maleic acid, 250 mM NaCl (pH 7.5)]. Slides were heated to 80°C for 3  min and incubated in a humidified chamber for 150 min in the dark at RT. Slides were washed twice for 15 min each in 1× PBS containing 30% (v:v) formamide, 10 mM Tris–HCl, and 1% (w:v) BSA, and three times for 5 min each in TBS buffer containing 20 mM Tris–HCl, 150 mM NaCl, and 0.1% (v:v) Tween-20. Finally, immunofluorescence of γ-H2A.X *foci* was performed on PNA-hybridized cells as described: coverslips were blocked for 1 h/RT with PBS containing 1% (w:v) BSA, 0.5% (w:v) fish gelatine, and 0.1% (v:v) Tween-20. Thereafter, coverslips were incubated overnight with rabbit monoclonal anti-γ-H2A.X (2.8 ng/μL, phospho-S139 – clone EP854(2)Y; Abcam) in a humid chamber at 4°C. After washing with 1× PBS, sensitive detection was achieved through an anti-rabbit Alexa Fluor™ 488 (5 ng/μL; Thermo Fisher Scientific) as a secondary fluorochrome-labelled antibody (1 h/RT). Coverslips were mounted into glass slides using mounting media containing DAPI (5 ng/μL). Images were acquired with a confocal microscope (Zeiss LSM 980) using a ×63 magnification objective (Nikon). Telomere spots were quantified with Fiji software using the Find Maxima tool with variable values of prominence depending on each experiment: each cell was analysed, and quantification (number of *foci* identified) made by the software was also checked and confirmed by eye-inspection. Co-localization rate of telomere spots with γ-H2A.X *foci* was quantified by eye-inspection considering the sum of green (γ-H2A.X) and red (TelG-Cy3) resulting in a yellow signal (co-localization) as G4-forming telomeres and damaged telomeres, respectively.

### G4 stabilization assays

The following DNA sequences were synthesized and used for circular dichroism (CD) experiments: d(TTAGGGTTAGGGTTAGGGTTAGGGTT) (Tel_26_), d(GGCTTAGGCTTAGGCTTAGG) (Ce20). The DNA sequences were synthesized using standard β-cyanoethyl phosphoramidite solid phase chemistry at the 1 μmol scale and purified as described elsewhere [46]. Telomeric G4s were prepared in 5 mM KH_2_PO_4_/K_2_HPO_4_ buffer (pH 7.0), containing 20 mM KCl. Tel_26_ G4 in the hybrid arrangement (Tel_26-hy_) was obtained by preparing the sample at a single-strand DNA concentration of 0.5 mM, while the parallel arrangement (Tel_26-p_ G4) was obtained using a single-strand DNA concentration of 10 mM [47–49]. Ce20 G4, instead, was prepared in 20 mM KH_2_PO_4_/K_2_HPO_4_ buffer (pH 7.0), containing 70 mM KCl. All samples were heated at 90°C for 5 min and then gradually cooled to RT overnight, and stored for 24 h, before data acquisition. All DNA samples were diluted to 2 μM before CD experiments. DNA concentration was verified by measuring the ultraviolet absorption at 90°C, considering the appropriate molar extinction coefficient values ϵ (λ = 260 nm), calculated as described elsewhere [50]. CD experiments were performed on a Jasco J-815 spectropolarimeter equipped with a PTC-423S/15 Peltier temperature controller. CD spectra of the G4-forming oligonucleotides and of the G4/ligand mixtures, obtained by adding 2 mol equivalent of ligand, were recorded at 20°C in the 230- to 320-nm wavelength range, using 1 mm path-length cuvettes. The scan rate was 100 nm/min with a 0.5 s response time and bandwidth of 1 nm. Spectra were averaged over three scans. Buffer baseline was subtracted from each spectrum. CD melting experiments were recorded in the 20°C–100°C temperature range at a 1°C/min heating rate by following changes of the CD signal at the wavelengths of the maximum CD intensity (i.e. 264 nm for Tel_26-p_ G4, 289 nm for Tel_26-hy_ G4, and 292 nm for Ce20). CD melting experiments were performed in the absence and presence of compounds (2 molar equivalent). Apparent melting temperatures (*T*_m_) were determined by using a non-linear curve fitting with the Boltzmann function curve fit on OriginLab^©^ 2021 software (OriginLab Corp., MA, USA).

### FANCJ protein expression and purification

The human FANCJ DNA helicase was produced in HEK 293T cells, transiently transfected with plasmid pCSII-EF-MCS (version 3.4) harbouring Flag-tagged full-length FANCJ open reading frame (a gift from Hisao Masai, Tokyo Metropolitan Institute of Medical Science, Tokyo, Japan) [51]. The FANCJ recombinant protein was purified as previously described [43].

### Gel-based helicase activity assay

DNA oligonucleotides were purchased from Biomers (Ulm, Germany) and listed in Supplementary Table S2. G4 DNA structures were formed by heating mixtures containing each indicated oligonucleotide at a concentration of 1 μM in annealing buffer [10 mM Tris–HCl, 1 mM EDTA, 100 mM KCl (pH 7.5)] for 2 min at 90°C. Then, each mixture was subjected to slow cooling (90 s/1°C) up to 10°C to allow annealing. FANCJ at the indicated concentrations was incubated in reaction mixtures (volume: 20 μL) containing each indicated FAM-labelled G4 DNA substrate (10 nM) in buffer H [25 mM HEPES–NaOH (pH 7.2), 100 mM KCl, 5 mM MgCl_2_, 2 mM DL-dithiothreitol (DTT), 0.01% (w:v) BSA] for 2 min at RT to allow the protein–DNA complex to form. G4 resolution was initiated by adding ATP (2 mM) together with an excess of a capture DNA oligonucleotide (200 nM) that hybridized with the sequence forming the G4 structure, thereby preventing it from reforming. After an incubation for 15 min at 30°C, the reactions were quenched with the addition of 5 μL of 5× Stop Solution [0.5% (w:v) sodium dodecyl sulfate (SDS), 40 mM EDTA, 0.5 mg/mL (w:v) proteinase K, 20% (v:v) glycerol]. Samples were incubated for 5 min at 20°C and then, run on a 12.5% polyacrylamide-bis (19:1) gel in 1× Tris borate EDTA buffer (TBE) containing 100 mM KCl at a constant voltage of 90 V on ice. The individual bands were visualized on a ChemiDoc MP imager, and the band intensities were determined using Image Lab software (Bio-Rad Laboratories).

### Phenotype screening in *C. elegans*

Nematodes were cultured at 20°C on nematode growth medium (NGM) plates with *E. coli* (OP50) as food source according to standard methods [52]. The wild-type strain Bristol N2 and VC13 *dog-1 (*gk10) *C. elegans* strains were obtained from the *Caenorhabditis* Genetics Centre and Reverse Genetics Core Facility at the University of British Columbia, respectively. L4 hermaphrodite worms were individually transferred to 12-well plates containing NGM supplemented with increasing concentrations of G4 stabilizers, as specified in the legend of Fig. 8. Thereafter, the plates were incubated at 20°C for 48 h. Each worm was transferred onto a fresh identical plate to allow laying of fertilized eggs for 4 h. To measure embryonic lethality, eggs were scored 24 h after laying, and the ratio of unhatched to the total laid eggs was calculated. Larval arrests were monitored up to 72 h after egg laying.

### Immunostaining in *C. elegans* germline

Gonads of adult worms, fed on NGM-containing compound FIM-15 (at 20 μM) for 48 h, were dissected in M9 buffer [0.3% (w:v) H_2_PO_4_, 0.6% (w:v) Na_2_HPO_4_, 0.5% (w:v) NaCl, 1 mM MgSO_4_] on Poly-Lysine glass slides. The specimens were freeze-cracked in liquid nitrogen, sequentially immersed at −20°C in methanol, methanol/acetone (1:1), and acetone, and washed three times for 5 min in 1× PBS. Slides were blocked in 1% (w:v) BSA and 0.5% (w:v) fish gelatine in 1× PBS for 1 h/RT in a humid chamber. Slides were incubated overnight with BG4 single-chain antibody (1 ng/μL) in a humid chamber at 4°C. After washing with 1× PBS, sensitive detection was achieved through an amplified fluorescence signal generated by incubation for 1 h/RT with anti-FLAG antibody produced in rabbit (at 5 ng/μL; Sigma–Aldrich, Catalogue No. F7425); followed by incubation for 1 h/RT with anti-rabbit Alexa Fluor™ 488 (5 ng/μL; Thermo Fisher Scientific) as a tertiary fluorochrome-labelled antibody. Thereafter, slides were mounted using mounting media containing DAPI (at 5 ng/μL). Images were acquired with a confocal microscope (Zeiss LSM 980) using a ×40 magnification objective (Nikon).

### Statistical analyses

**Figure 2.**
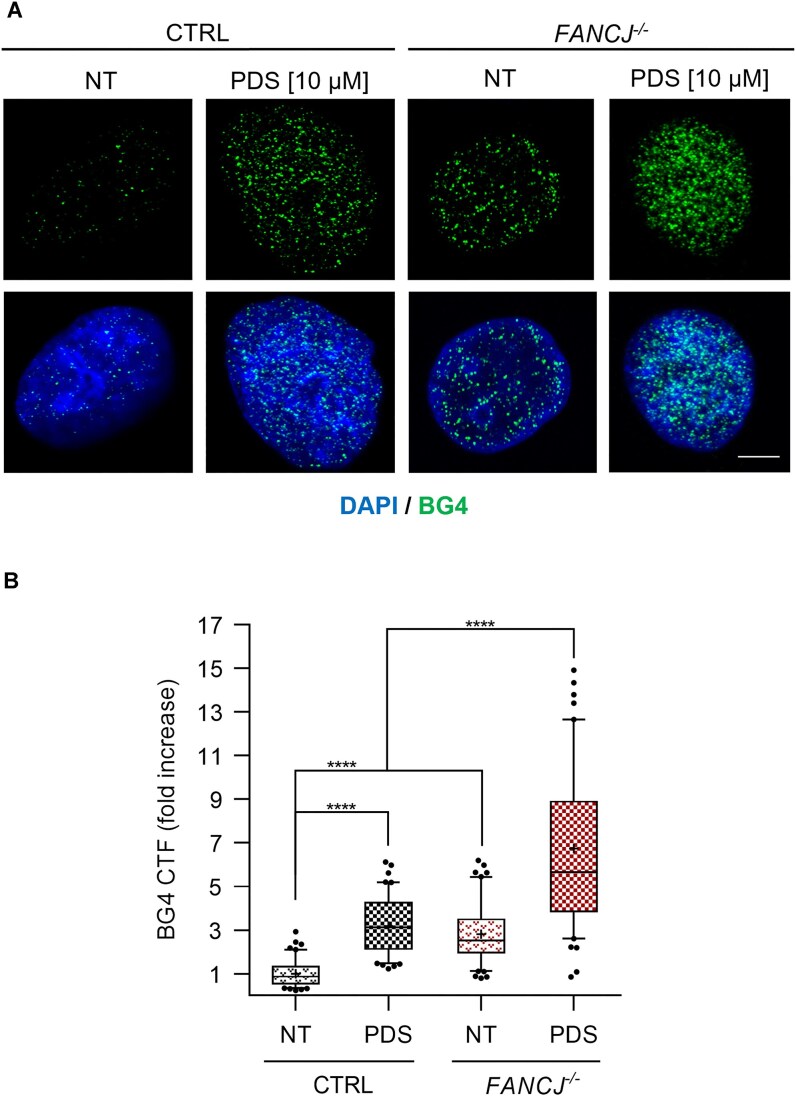
Analysis of G4 *focus* formation in *FANCJ*-KO HeLa cells. (**A**) Immunofluorescence showing BG4 *foci* in the indicated cell lines. HeLa cells were treated for 24 h with PDS (10 μM). Scale bar, 5 μm. (**B**) Fold increase of the BG4 CTF, detected per nucleus in the indicated cell lines and conditions. The chart reports medians (lines), mean (cross), 50% (box), and 90% (whiskers) of the datasets and outliers (circles). A total of 100 cells were analysed per condition in three biological independent experiments. Statistical analyses were performed using a non-parametrical Mann–Whitney *U*-test. Two-tailed *P*-values were calculated (****P < .0001).

**Figure 3.**
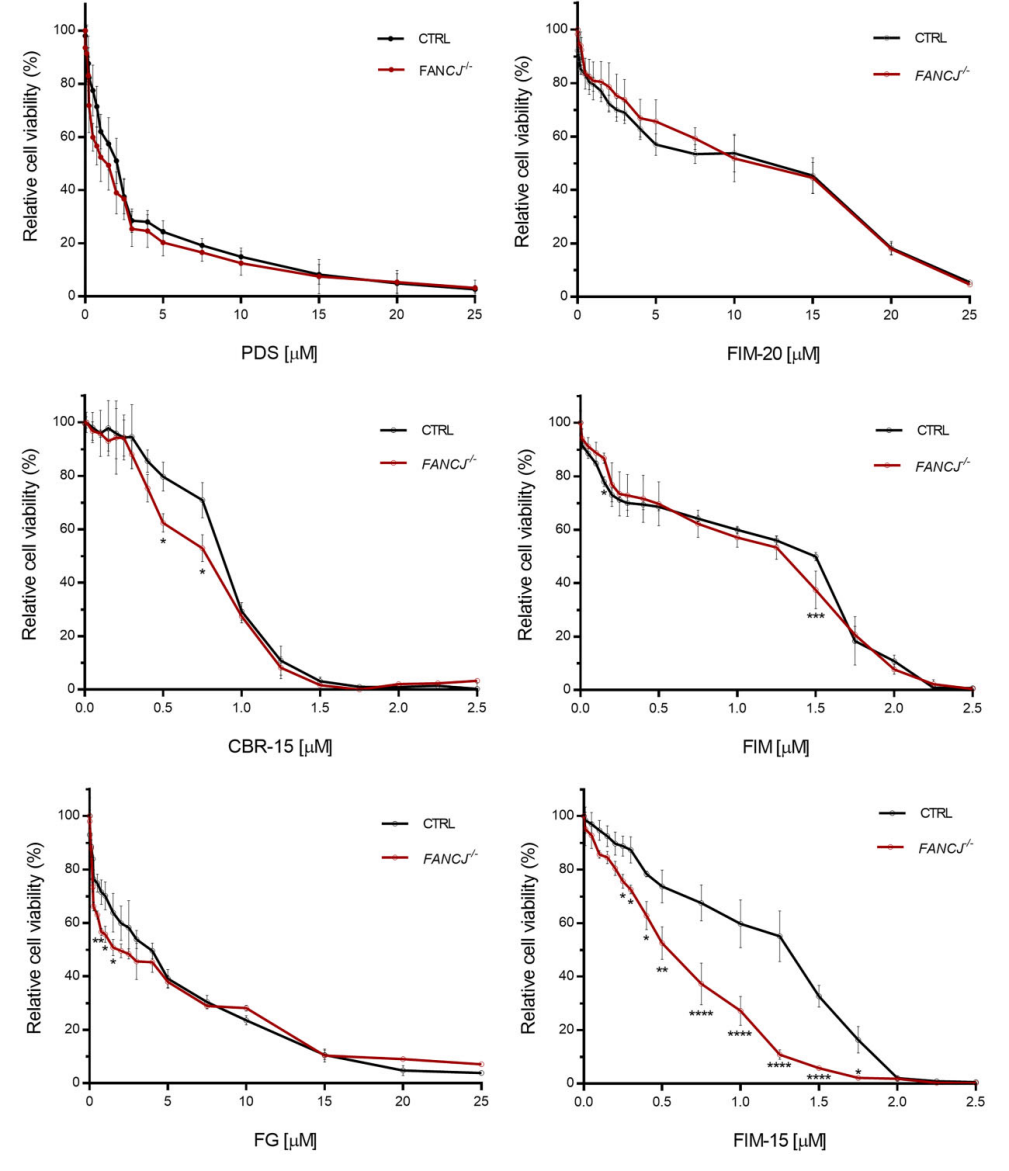
Effects of G4 stabilizers on viability of *FANCJ*-KO cells. Relative cell viability of control (CTRL) and *FANCJ*-KO (*FANCJ^−/−^*) HeLa cell lines after a 72-h treatment with the indicated G4 binders. Error bars show average ± SEM of three biological independent experiments. Data were analysed using one unpaired *t*-test per condition and corrected with the Holm–Šídák method. Reported *P*-values are *P < .05; **P < .01; ***P < .001; ****P < .0001.

**Figure 4.**
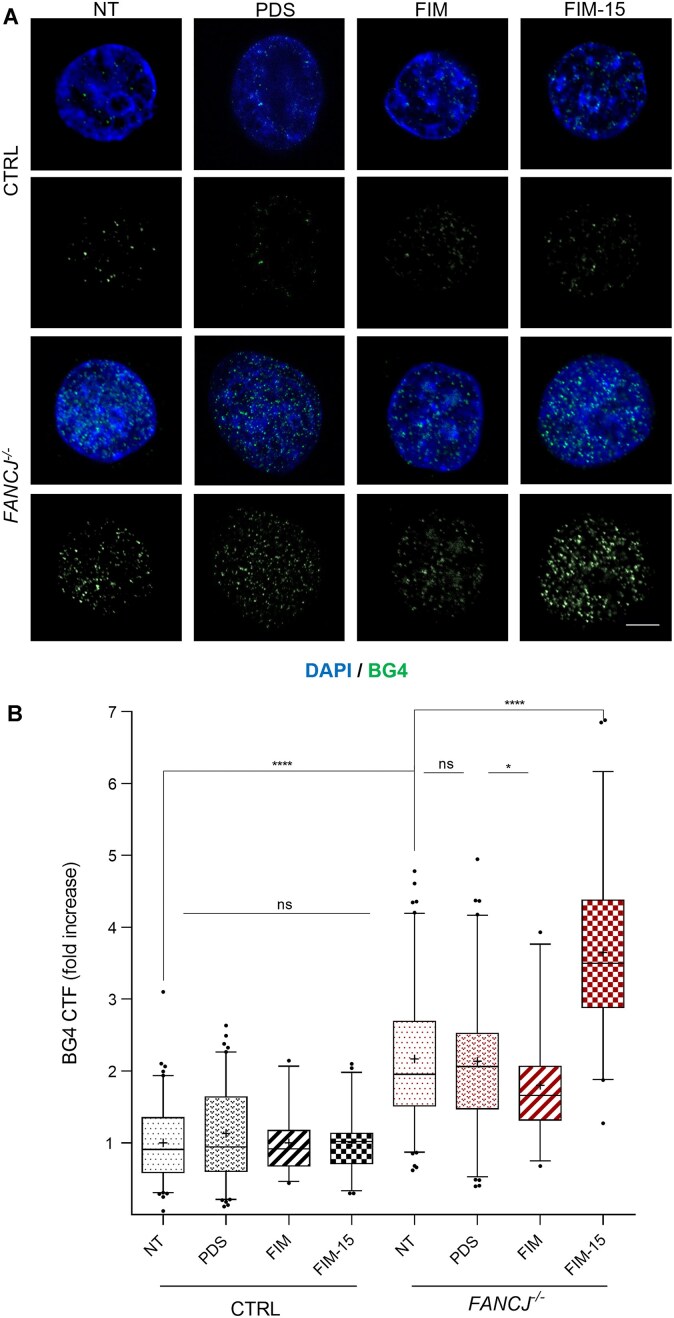
Quantitative analysis of G4 stabilization in *FANCJ*-KO cells by different G4 binders. (**A**) Immunofluorescence representative images showing BG4 *foci* in the indicated cell lines after treatment with PDS (500 nM), FIM (500 nM), and FIM-15 (250 nM) for 24 h. Scale bar, 5 μm. (**B**) Fold increase of the BG4 CTF, detected per nucleus in the indicated cell lines and conditions. The chart reports medians (lines), mean (cross), 50% (box), and 90% (whiskers) of the datasets and outliers (circles). A total of 100 cells were analysed per condition in three biological independent experiments. Statistical analyses were performed using a non-parametrical Mann–Whitney *U*-test. Calculated *P*-values were indicated as *P < .05; ****P < .0001.

**Figure 5.**
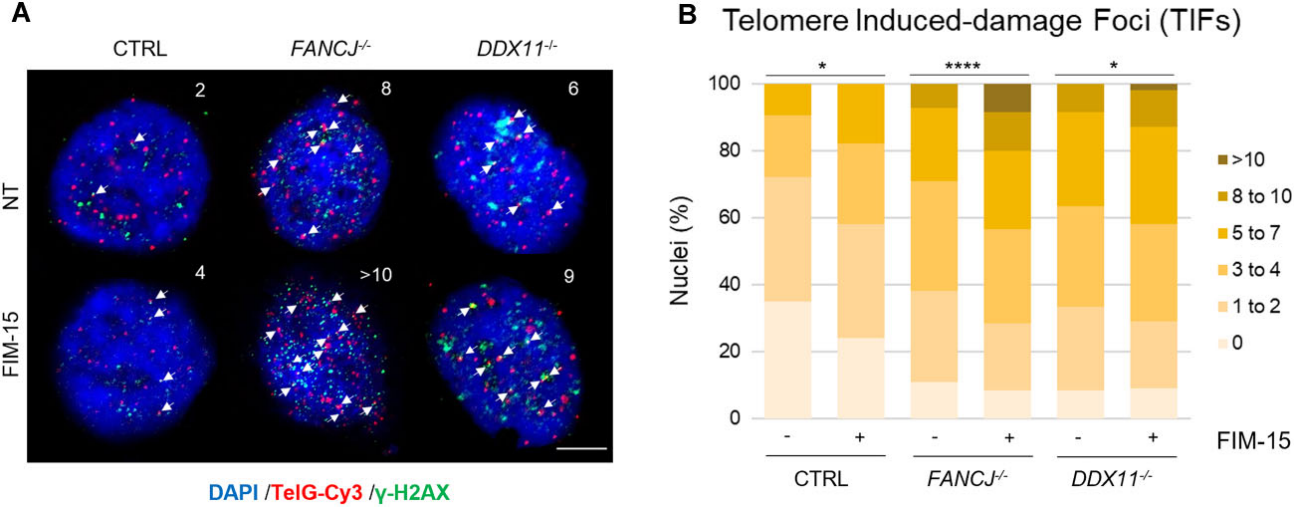
FIM-15 treatment enhances telomere damage in *FANCJ*-KO cells. (**A**) Co-localization of telomeres (TelG-Cy3 FISH) and damaged DNA (γ-H2A.X immunofluorescence) in interphase nuclei of the indicated cell lines, treated with FIM-15 (250 nM) for 24 h. Arrows indicate TIFs (sites of signal co-localization). Scale bar, 5 μm. (**B**) Percentage of nuclei with indicated number of TIFs was determined for at least 100 cells in each experiment in three biological replicates. Statistical analyses were performed using a parametric Student’s *t*-test for unpaired data. Reported *P*-values were *P < .05; ****P < .0001.

Statistical analyses to compare BG4 CTF in Figs 2 and 4 were performed using a non-parametrical Mann–Whitney *U*-test. Two-tailed *P*-values were calculated, comparing two groups at a time. Experiments in Figs 3 and 8 were analysed following a significant effect reported in unpaired *t* multiple comparison test per condition to compare treatments and corrected with the Holm–Šídák method. Statistical analyses in Fig. 5 were performed using a parametric Student’s *t*-test for unpaired data.

## Results

### Effects of hydrazone derivatives on viability, G4 stabilization, and DNA damage induction in *FANCJ*-KO HeLa cells

To capture the incidence of G4 DNA structures in HeLa cells, we used the well-characterized anti-G4 DNA antibody, BG4 [53], produced and purified as described in the ‘Materials and methods’ section. As shown in Fig. 2A, HeLa cells exhibited a distinct punctate nuclear staining, indicative of G4 DNA structures formation. Notably, following a 24-h treatment with PDS, a well-recognized G4-stabilizing agent, the number of G4 *foci* appeared to be enhanced (Fig. 2). We analysed G4 *focus* formation in *FANCJ*-KO cells and, in line with the important role reported for the FANCJ DNA helicase in dismantling G4 DNA structures in metazoans [28–32], we found that the number of G4 *foci* was strikingly increased in this line compared with the isogenic control one (Fig. 2). Following treatment with PDS, the CTF confirmed a heightened level of G4 *foci* in FANCJ-depleted cells compared with the control line (Fig. 2B).

Then, we tested the impact of FANCJ deficiency on viability of HeLa cells upon 72-h treatment with different G4 stabilizers, including PDS, three mono- and one bis-guanylhydrazone derivatives of diimidazo[1,2-a:1,2-c]-pyrimidine (FIM, FIM-15, FIM-20, and FG, respectively; Fig. 3) [24], as well as a mono-hydrazone analogue linked to a benzene ring which in turn is substituted with an indolinone unit (CBR-15; Fig. 1) [22]. Cell cultures were exposed to increasing concentrations of these compounds and viability was measured using a colorimetric assay, based on the reduction of XTT to an orange formazan product in living cells, as described in ‘Materials and methods’ section. We found that *FANCJ*-KO HeLa cells displayed a viability comparable to the control line, when treated with all the aforementioned G4 ligands with the only exception of the FIM-15 compound (see Fig. 3 and Supplementary Table S1) [24]. Of note, FANCJ-deficient HeLa cells were found to be highly sensitive to treatment with FIM-15, even when this G4 ligand was administered in the nanomolar range.

Thereafter, immunofluorescence experiments to detect G4 *foci* were carried out following 24-h treatment with a subset of the aforementioned G4 stabilizers at low doses. As reported in Fig. 4, the most remarkable effect on G4 *focus* formation was observed upon treating *FANCJ*-KO cells with the FIM-15 compound (250 nM). A very small but significant decrease in the BG4 immunofluorescence signal was observed when *FANCJ*-KO cells were treated with FIM. However, the distribution of the BG4 signals per nucleus falls within the range observed for the untreated and PDS-treated conditions. Then, we went on analysing the effect of FIM-15 on DNA damage induction in *FANCJ*-KO cells by immunofluorescence experiments using a monoclonal antibody that specifically recognizes the γ-H2A.X phosphorylated histone variant. We found that, in the absence of FANCJ, cells treated with FIM-15 displayed an increased number of γ-H2A.X *foci*, indicative of enhanced DNA damage (Supplementary Fig. S1).

### Treatment with FIM-15 impairs telomere integrity in *FANCJ*-KO HeLa cells

Next, we analysed telomere integrity in *FANCJ*-KO HeLa cells upon treatment with FIM-15 (250 nM). This analysis was also extended to a HeLa cell line, where the gene coding for the DDX11 DNA helicase was knocked-out (*DDX11*-KO HeLa) [44]. DDX11 is a SF2 Fe–S cluster DNA helicase that shares sequence similarity with FANCJ. It plays a key role in coupling DNA replication with sister chromatid cohesion establishment [54]. Moreover, DDX11 is believed to counteract DNA replication stress that derives from formation of G4 and other DNA secondary structures that impede the smooth progression of the DNA replication machinery [44, 55, 56]. Initially, we examined the presence of G4 DNA structures at telomeres in interphase cell nuclei by combining immunofluorescence with the BG4 antibody and fluorescence *in situ* hybridization (FISH) with a Cy3-labelled telomeric probe. The results of this analysis revealed that approximately half of the telomeres, detected by FISH, co-localized with G4 *foci*, either in control or in FANCJ- (and DDX11-) depleted HeLa cells (Supplementary Fig. S2). Our findings suggest that G4 DNA structures are largely present in interphase nuclei outside telomeres. Moreover, while chromosomal ends might have a propensity to form different G4s, our results might also be explained by differences in antibody accessibility to these structures (e.g. masking by telomere-binding proteins such as components of the protective Shelterin complex). Furthermore, despite observing an increased number of BG4 *foci* in mutant cell lines lacking either FANCJ or DDX11, compared with the control line (as shown in Fig. 2), the proportion of G4 structures co-localizing with telomeres remained unchanged in the different genetic backgrounds. This suggests that the absence of any of these DNA helicases did not directly affect the observed incidence of G4s localized at chromosomal ends. To gain deeper insights into the impact of FANCJ or DDX11 loss on telomeric G4 metabolism, additional experiments were carried out to assess telomere dysfunction-induced *foci* (TIFs). In these experiments, immunostaining with an anti-γ-H2A.X antibody was combined with telomeric FISH to visualize DNA damage at chromosomal ends in interphase nuclei. HeLa cell lines were subjected to a 24-h treatment with low doses of FIM-15, followed by TIF detection and quantification in the different conditions (Fig. 5). A substantial increase of nuclei with dysfunctional telomeres was observed in the two mutant lines lacking FANCJ or DDX11 compared with the control nuclei. Treatment with FIM-15 induced a higher number of TIFs in each cell line compared with the respective untreated condition. Notably, upon administration of FIM-15, *FANCJ*-KO cells displayed an enhanced number of nuclei with dysfunctional telomeres compared with both control and *DDX11*-KO lines.

Overall, these results suggest that the FANCJ DNA helicase plays a pivotal role in dismantling G4 DNA structures stabilized by FIM-15, a guanylhydrazone derivative, in different chromosomal sites, including chromosomal ends. When FANCJ was absent, stabilization of the G4 DNA structures by FIM-15 enhanced DNA damage and reduced cell viability.

### *In vitro* G4 stabilization experiments

To explain the effects of FIM-15 in *FANCJ*-KO cells compared with other G4 ligands, we assessed their ability to bind and stabilize G4 structures formed by G-rich at human chromosomal ends. Various G4 topologies can be found at human telomeres, depending on sequence context and local environmental conditions [57]. In this study, the 26-nt sequence (Tel_26_) was examined, which forms a hybrid [3 + 1] G4 conformation (Tel_26-hy_) in K^+^-containing solution. Given evidence that the predominant telomeric G4 conformation under crowded cellular conditions is parallel [58], we prepared a high-concentration Tel_26_ sample to promote the parallel fold (Tel_26-p_) [47–49]. The stabilizing effects of PDS, PhenDC3, CBR-15, FIM, FG, FIM-15, and FIM-20 on the two G4 topologies adopted by Tel_26_ DNA were investigated using CD melting experiments, which measured the compound-induced change in the apparent melting temperature (Δ*T*_m_) of G4s. Results (Table 1) revealed that all compounds generally stabilized the parallel G4 conformation (Δ*T*_m_ $ \ge$ 14°C) more effectively than the hybrid topology (Δ*T*_m_≤ 10°C) adopted by Tel_26_ sequence, with the exception of PhenDC3, which exhibited no preference between the two topologies. Interestingly, FIM-15 was the only hydrazone derivative that significantly stabilized the Tel_26-hy_ conformation. Although PDS and PhenDC3 showed greater stabilizing effects on Tel_26-hy_ conformation compared with FIM-15, they failed to exhibit any increased cytotoxic effects in FANCJ-depleted cells compared with the isogenic control line. This discrepancy may be attributed to differences in the binding modes of FIM-15, PDS, and PhenDC3 to G4s in the nuclear compartment, which could distinctly influence cellular response.

**Table 1.** Ligand-induced thermal stabilization of Tel_26_ G4s measured by CD melting experiments

Δ*T*_m_ (°C)^1^		
Compound	Tel_26-hy_ G4	Tel_26-p_ G4
PDS	+10.0	>20
PhenDC3	>20	>20
FG	−4.5	>20
FIM	−3.0	+18.0
CBR-15	+1.0	>20
FIM-20	+1.8	+14.0
FIM-15	+4.3	>20

^1^ΔTm (± 0.5 °C) represents the difference in melting temperature [ΔTm = Tm (DNA + ligand (1:2)) − Tm (DNA)].

### Resolution of unimolecular G4 DNA structures with different topology by the FANCJ DNA helicase

**Figure 6.**
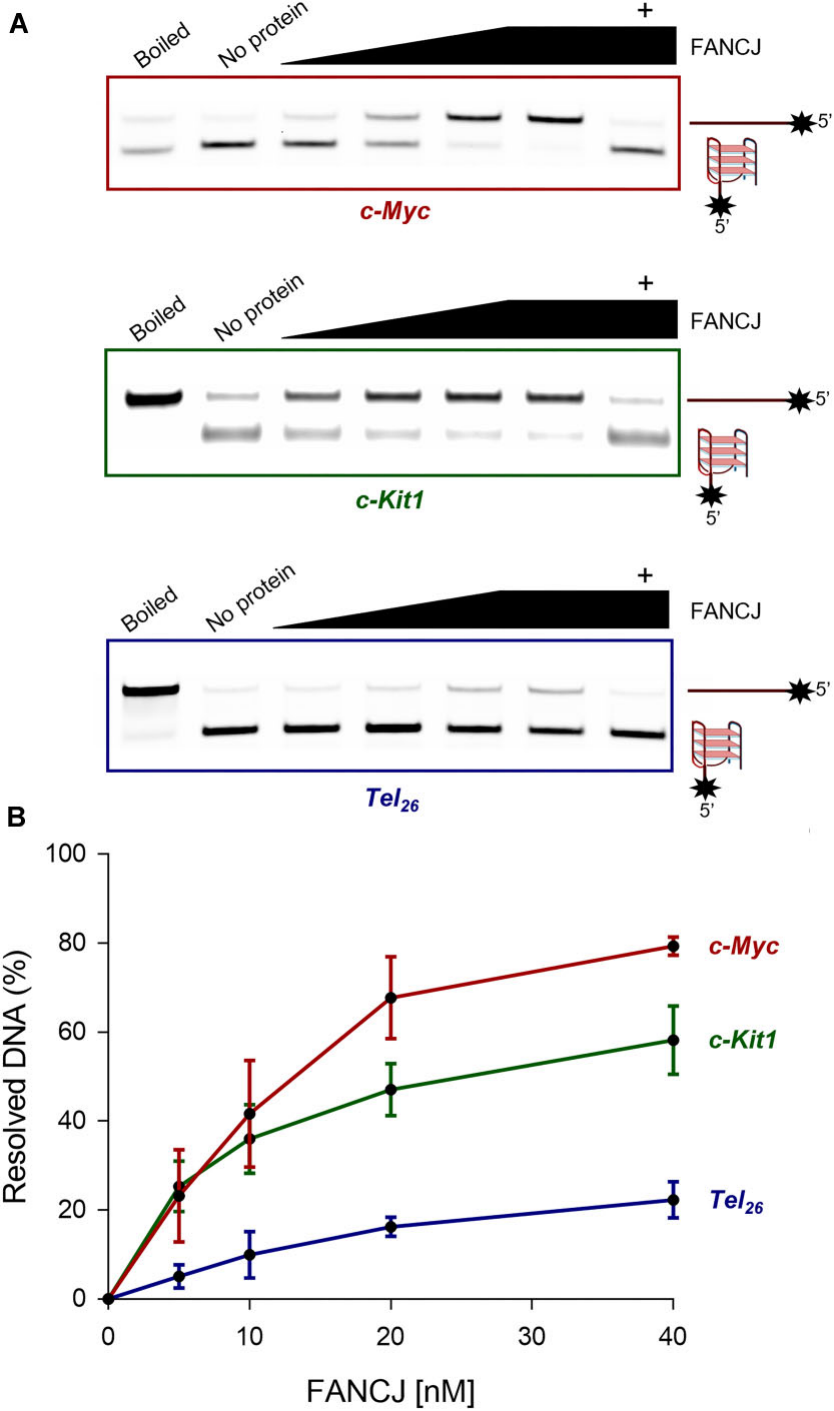
FANCJ gel-based DNA helicase assays. (**A**) FANCJ helicase activity assays were carried out using increasing concentrations (0, 5, 10, 20, and 40 nM) of the recombinant protein with the indicated fluorescent-labelled unimolecular G4 DNA substrates. Lane, named ‘Boiled’ in each gel, contains a heat-denatured assay mixture with no protein. Lane, named ‘No protein’ in each gel, contains a mock assay without FANCJ. Lane, named ‘+’ in each gel, refers to an assay carried out in the presence of FIM-15 (1 μM) with the highest concentration of FANCJ protein (40 nM). (**B**) Graph reports data of three independent assays (mean  ±  SD) with the indicated G4 DNA substrates.

Previous biochemical studies revealed that human FANCJ efficiently resolves unimolecular G4 DNA structures in a ATPase-dependent manner, whereas other Fe–S DNA helicases (such as DDX11 and XPD) did not display this ability, when tested by *in vitro* gel-based DNA helicase assays [30]. Thus, we decided to assess preference of human FANCJ in unwinding unimolecular G4s with different topologies. Full-length Flag-tagged FANCJ recombinant protein, purified from transiently transfected mammalian cells (see ‘Materials and methods’ section and Supplementary Fig. S3A), was pre-incubated with different unimolecular G4 DNA structures prepared from fluorescent-labelled G-rich oligodeoxynucleotides. Then, G4 unfolding was started by adding ATP to the reaction mixture. An unlabelled single-stranded oligonucleotide (DNA trap) complementary to the G4-forming sequence was also added to the reaction mixture to prevent G4 refolding. Reaction products were separated on a polyacrylamide gel exploiting the higher mobility displayed by the more compact G4 structure. As shown in Fig. 6, FANCJ resolved unimolecular parallel G4 DNA structures (such as the ones derived from the c-*Myc* and c-*Kit1* sequences) with higher catalytic efficiency compared with the telomeric hybrid structure formed by the *Tel*_26_ DNA oligonucleotide (see Supplementary Table S2 for the oligonucleotide sequences). Moreover, when control DNA helicase assays were carried out on the aforementioned unimolecular G4 substrates in the presence of FIM-15 (1 μM) with the highest FANCJ concentration (40 nM), a complete inhibition of the helicase activity was observed (last band in each gel of Fig. 6A). Further assays carried out, titrating FIM-15 concentration, showed a gradual inhibition of the FANCJ DNA helicase activity with all three G4 DNA substrates tested (Supplementary Fig. S3B). This suggests that the FIM-15 hydrazone derivative inhibits the FANCJ resolving activity by binding specifically to the G4 DNA structure that becomes not any more accessible to the enzyme (Fig. 6A). In fact, the FANCJ catalytic function was not inhibited by FIM-15 in assays carried out on a forked duplex DNA (Supplementary Fig. S3C). Taken together, these results indicate that FANCJ has a clear preference for the unimolecular G4 DNA structures with a parallel topology, like those found in gene promoter regions, compared with the telomeric hybrid G4s. These findings are also consistent with the G4-binding and stabilizing properties described for FIM-15, which preferentially binds and stabilizes the unimolecular parallel G4 DNA structures, as the one formed by the G-rich DNA sequences of the c-*Kit* and c-*Myc* promoter regions, over hybrid topologies formed by the G-rich sequences found at chromosome telomeric ends [24].

### Effect of FIM-15 in a *C. elegans dog-1^−/-^* strain

**Figure 7.**
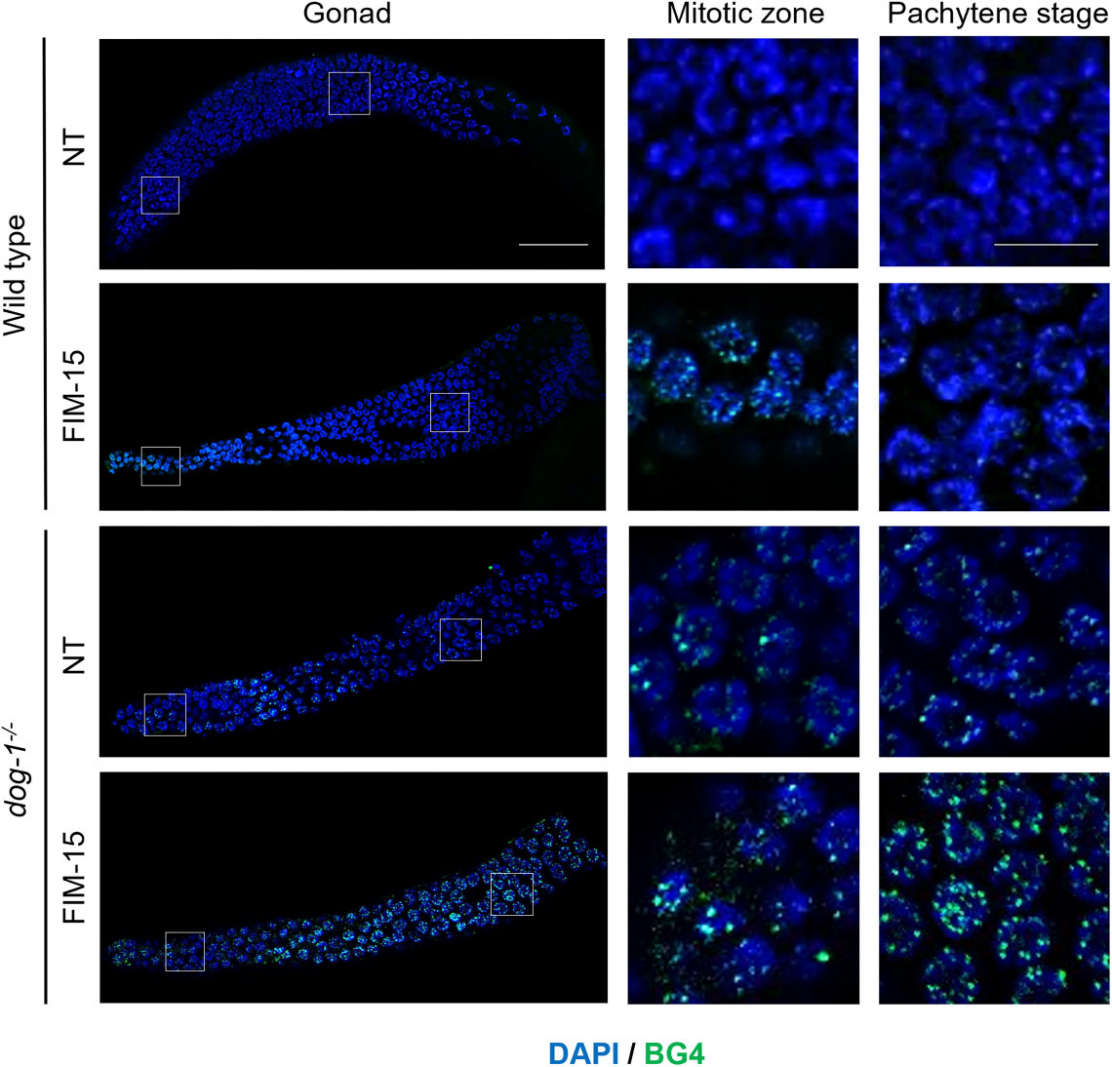
Detection of G4 *foci* in the gonadal nuclei of *C. elegans dog-1^−^^/^^−^*. The indicated worm strains were subjected to a 24-h treatment with FIM-15 (20 μM). Nuclei were co-stained with the BG4 antibody and DAPI. Scale bar, 20 μm. Insets, indicated by boxes, show sections of the main images corresponding to the mitotic zone and pachytene stage nuclei. Scale bar, 5 μm.

To study the effect of the FIM-15 compound in the context of a whole organism, we used the *C. elegans* model system. Disruption of the gene coding for DOG-1, the nematode ortholog of mammalian FANCJ, was reported to cause germline and somatic deletions upstream of genes containing poly-guanine tracts [28, 42]. This suggests a role of DOG-1 in resolving G4 DNA structures at G-rich worm genome *loci*. Initially, we analysed the incidence of G4 *foci* in *C. elegans* gonadal nuclei by immunofluorescence with the BG4 antibody. As shown in Fig. 7, G4 structures were detected in wild-type worm gonads, mainly after administration of the FIM-15 compound (20 μM). Of note, G4 *foci* were much more abundant in *dog-1^−/-^* compared with wild-type worm gonads, in either treated or untreated conditions.

**Figure 8.**
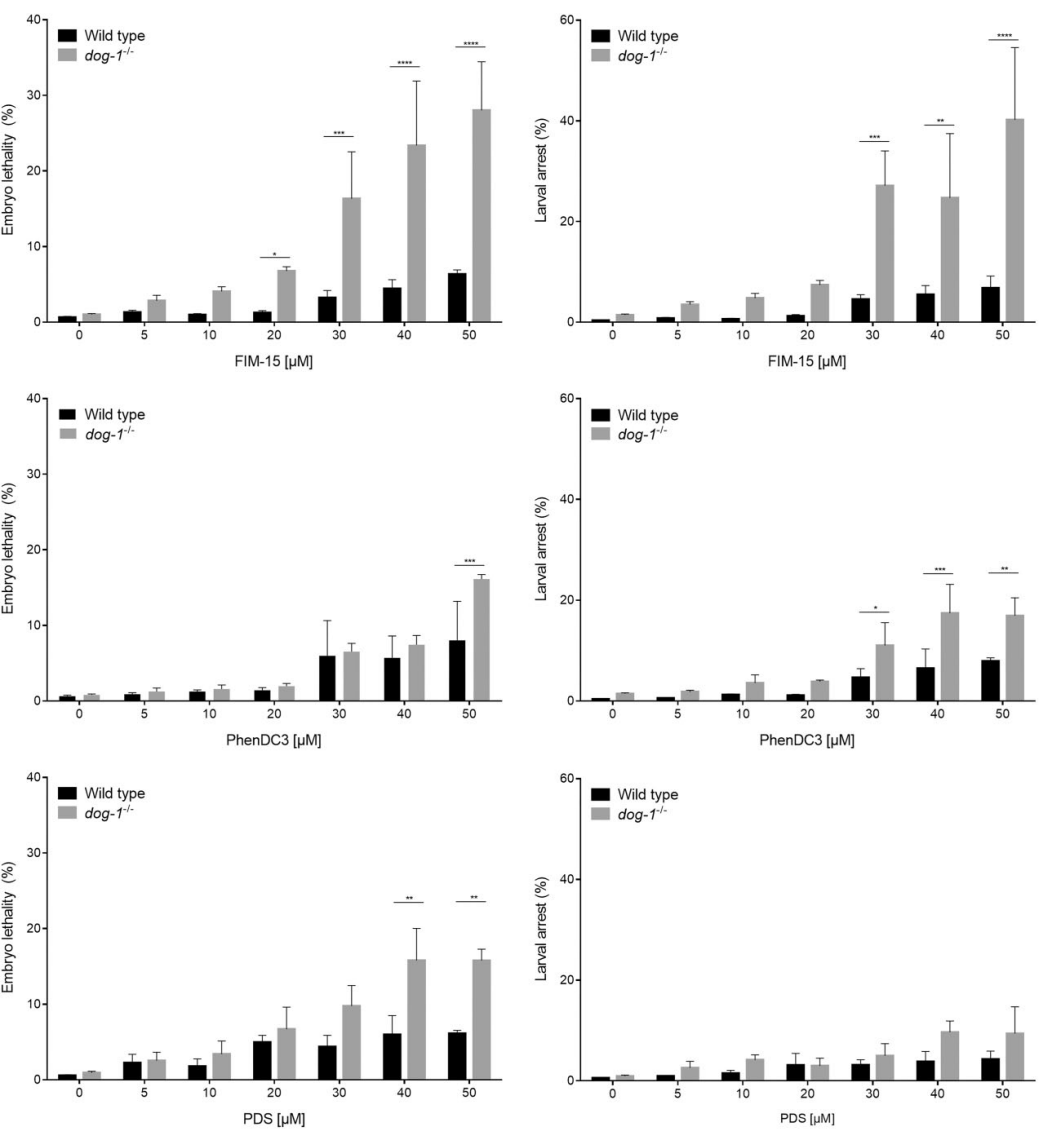
Effect of G4 binders on *dog-1^−^^/^^−^* worm development. Larval 4 nematodes were treated for 48 h with the indicated concentrations of FIM-15, PhenDC3, or PDS. Embryonic lethality and larval arrests were scored as ratio of unhatched to the total laid eggs and arrested larvae on total hatched eggs from isolated parental worms, respectively, for 4 h after treatment. At least 700 first-generation animals were analysed in each condition in three biological independent experiments. Data were analysed using one unpaired *t*-test per condition and corrected with the Holm–Šídák method. Reported *P*-values are *P < .05; **P < .01; ***P < .001; ****P < .0001.

Thereafter, we tested the effects of FIM-15 during worm development, using PhenDC3 and PDS, two well-known G4 stabilizers, as reference compounds. We found that all these G4 stabilizers, especially at the highest concentrations used, had an adverse impact on nematode development, as evidenced by compromised embryonic survival and presence of larval arrests in the first-generation post-treatment (Fig. 8). Moreover, our results revealed an enhanced susceptibility of the *C. elegans dog-1^−/−^* strain to all these compounds when compared with its wild-type counterpart. Nevertheless, in the absence of DOG-1, FIM-15 exerted its detrimental effects on the worm development, even at low micromolar concentrations, showing a higher incremental ratio compared with PhenDC3 and PDS.

Furthermore, to verify that FIM-15 was able to bind to G4 structures predicted to arise in the *C. elegans* genome, CD experiments were performed on the deoxy-oligonucleotide Ce20 that derives from the worm telomeric sequence [59]. Initially, the G4 conformation adopted by the Ce20 sequence was assessed using CD spectroscopy. The CD spectrum displayed a positive band at 292 nm and negative one at 264 nm, characteristic values of antiparallel-stranded G4s (Supplementary Fig. S4A) [60]. Notably, no change in the CD spectrum of the Ce20 G4 DNA structure was observed upon addition of FIM-15 (Supplementary Fig. S4A). The stabilizing effect of FIM-15 on the Ce20 G4 was assessed by evaluating ligand’s ability to induce G4 thermal stabilization (Δ*T*_m_). This was done by comparing CD-melting experiments recorded at 292 nm for the G4, both in the presence and absence of compound (Supplementary Fig. S4B). FIM-15 showed a good stabilizing effect on the Ce20 G4 (Δ*T*_m_ = 14.5°C). In addition, to provide a reference for a known G4 binder, we conducted the same experiments in the presence of PhenDC3 and PDS (Supplementary Fig. S4B), which showed a ligand-induced stabilizing effect >20°C.

### Discussion

G4s are emerging as important targets to discover novel anti-cancer drugs [61–63]. These unconventional DNA structures are expected to arise at G-rich genomic *loci*, especially during the genome duplication process, when single-stranded DNA regions are formed at the replication fork by the action of the CDC45/MCM2-7/GINS (CMG) complex, the replicative DNA helicase [32]. G4 structures represent strong roadblocks to the advancement of the DNA replication machinery [64]. In fact, although G4s arising on the leading strand are by-passed by the CMG complex with a molecular mechanism not yet fully understood [65], they were found to strongly stall DNA polymerase ϵ, inducing replication stress and activation of an ATR-mediated DNA damage response, if not promptly resolved. Dismantling these unconventional DNA secondary structures is the specific task of auxiliary DNA helicases (such as FANCJ, RTEL1, Bloom and Werner helicases, PIF1, and DHX36) that are recruited to the DNA replication machinery with different mechanisms [43, 66–69]. Therefore, preventing the activity of these specialized enzymes with compounds that specifically bind and stabilize G4 DNA structures is emerging as an innovative strategy to target highly proliferating cancer cells. Human FANCJ was demonstrated to be a very powerful G4 resolvase *in vitro* and is believed to play a prominent role in untangling G4 DNA structures at the DNA replication fork, where it is recruited by directly binding to AND-1/WDHD1, an evolutionarily conserved component of the mammalian replisome [30, 43, 70]. Moreover, FANCJ was found to be mutated with high frequency in breast and ovarian cancers [34], as well as in prostate [35] and colon cancer [36]. Thus, compounds that bind and stabilize G4 DNA structures are expected to sensitize *FANCJ*-defective cancer cells. In this study, we tested two well-known G4 ligands, PDS and PhenDC3, together with a set of G4-targeting guanylhydrazone-based molecules in *FANCJ*-KO HeLa cells. Specifically, we chose CBR-15, FG, FIM, FIM-15, and FIM-20, since, in previous studies, these compounds exhibited the highest cytotoxic potency in human cancer cell lines, such as U2OS and HeLa [22, 24]. We found that FIM-15 reduced viability of the *FANCJ*-KO HeLa line more than the isogenic control counterpart. In addition, treating *FANCJ*-depleted cells with FIM-15 at low doses enhanced the number of G4 *foci* and dysfunctional telomeres. A similar, but less exacerbated, phenotype was observed in DDX11-depleted HeLa cells treated or not with FIM-15, suggesting a possible involvement of DDX11 in metabolism of G4 DNA structures, including the ones at chromosomal ends.

It would be interesting to examine and compare CX-5461 and QN-302, the G4 ligands that are currently in clinical trials [71–73], with the hydrazone-based compounds used in this study. CX-5461 was reported to induce selective killing of homologous-recombination-deficient (HRD) tumours in preclinical models. However, it was recently demonstrated that CX-5461 is a potent mutagen that causes extensive genetic changes in different HRD-proficient (or not proficient) human cell lines, rising substantial safety issues [74, 75]. While, on one hand, CX-5461 was reported to hardly affect viability of *FANCJ*-KO RPE-1 cells [56], on the other hand, the QN-302 compound was been tested on HRD mammalian cell lines, to our knowledge.

In a previous biochemical study, FANCJ was reported to dismantle different G4s, such as uni-, bi- and tetra-molecular structures. Notably, FANCJ was found to be unique among the Fe–S DNA helicases for its ability to untangle unimolecular G4 DNA structures with very high catalytic efficiency [30]. Here, we report for the first time that unimolecular G4s with a parallel topology (such as the ones formed by the c-*MYC* and c-*KIT1* oligonucleotides) are resolved by the human FANCJ DNA helicase with higher catalytic efficiency compared with the hybrid one (formed by the *Tel*_26_ oligonucleotide in the helicase assay condition). These findings are consistent with the effects exerted in FANCJ-depleted HeLa cells by FIM-15, which was found to preferentially bind and stabilize parallel G4 DNA structures *in vitro* (see [24] and Table 1).

Notably, for the first time, we leveraged *C. elegans* to test the effect of dietary delivered G4 stabilizers in a whole organism. We were able to detect an enhanced number of G4 *foci* in *dog-1^−/−^* worm gonadal germ cells after FIM-15 administration. Moreover, an adverse impact on embryonic survival and larval development was scored in FIM-15-treated *dog-1^−/−^* nematodes. Our results open to the possibility of using *C. elegans* as a simple model system to screen new G4 binders in medicinal chemistry. Overall, our results pave the way for future therapeutic strategies targeting G4 DNA structures in *FANCJ*-defective tumours using the FIM-15 guanylhydrazone derivative.

## Supplementary Material

zcaf004_Supplemental_File

## Data Availability

The authors confirm that the data supporting the findings of this study are available within the article and its supplementary materials. Raw data and derived data supporting the findings of this study are available from the authors on request.
