# Supplementary material for: Effects of hydrazone-based G-quadruplex ligands on FANCJ/BRIP1-depleted cancer cells and a Caenorhabditis elegans dog-1−/− strain
Source: NAR Cancer. 2025 Feb 8;7(1):zcaf004. doi: 10.1093/narcan/zcaf004 (PMC11806260; doi:10.1093/narcan/zcaf004)
Supplement: zcaf004_Supplemental_File [file zcaf004_supplemental_file.pdf]

Supplementary Data

Materials and methods

Immunofluorescence - To analyse γ-H2A.X *foci*, HeLa cells were grown for 24 h on coverslips in 6-well plates (3 × 10^5 cells/well) in absence or presence of G4-stabilisers as indicated in the figure legend. Coverslips were fixed in cold methanol for 8 min on ice and further blocked for 1 h at RT with PBS containing: 1 % (w:v) BSA, 0.5 % (w:v) fish gelatine, 0.1 % (v:v) Tween 20. Then, coverslips were incubated overnight with rabbit monoclonal anti-γ-H2A.X (2.8 ng/µL, phospho-S139 - clone EP854(2)Y, Abcam) in a humid chamber at 4 °C. After washing with PBS 1x, coverslips were incubated for 1 h at RT with goat anti-rabbit Alexa Fluor™ 488 (5 ng/µL, ThermoFisher Scientific) as a secondary antibody. Thereafter, coverslips were mounted into glass slides using mounting media containing DAPI (5 ng/µL). Images were acquired with a confocal microscope (Zeiss LSM 980) using a × 63 magnification objective (Nikon). γ-H2A.X *foci* were quantified with Fiji software using the following formula to calculate the corrected total fluorescence (CTF): CTF = Integrated Density – (Area of selected nuclei x Mean fluorescence of background readings).

Fluorescence *in situ* hybridization (FISH) analysis - HeLa cells were grown 24 h on coverslips in 6-well plates (3 × 10^5 cells/well). Coverslips were incubated in cytoplasmic extraction buffer (20 mM HEPES-KOH, pH 7.9, 20 mM NaCl, 5 mM $MgCl_2$, 30 mM sucrose and 0.5 % [v/v] NP-40) for 10 min. Slides were washed very gently once with PBS 1x containing 0.1 % (v:v) Tween-20, once with PBS 1x and then fixed in cold Carnoy fixative (3:1 [v:v] methanol/acetic acid) for 8 min on ice. Slides were washed three times in PBS 1x, then incubated in pre-warmed pepsin solution (1 mg/mL in PBS 1x) 5 min at 37 °C. Thereafter, a fixation step was carried out with a solution containing 2 % (v:v) paraformaldehyde for 2 min at room temperature. After three washing steps in PBS 1x, coverslips were treated for 90 min at 37 °C with RNase A (at 50 µg/mL in PBS 1x supplemented with 0.1 M Glycine. Coverslips were washed in PBS 1x, then dehydrated with 70 %, 90 % and 100 % ethanol (2 min each), followed by air-drying. Thereafter, slides were overlaid with 50 nM of TelG-Cy3 PNA (Cy3-OO-KKK-ttagggttagggtt) in hybridisation buffer (10 mM Tris-HCl, 70 % [v:v] formamide, 1x Roche blocking reagent diluted in 100 mM maleic acid and 250 mM NaCl, pH 7.5). Slides were heated to 80 °C for 3 min and incubated in a humidified chamber 150 min in the dark at RT. Slides were washed twice for 15 min each in PBS 1x containing 30 % (v:v) formamide, 10 mM Tris HCl, 1% (w:v) BSA, and three times for 5 minutes each in TBS buffer containing 20 mM Tris HCl, 150 mM NaCl, 0.1 % (v:v) Tween 20. Finally, immunofluorescence of BG4 *foci* was performed on PNA hybridised cells as described: coverslips were blocked for 1 h at RT with PBS containing: 1% (w:v) BSA, 0.5% (w:v) fish gelatine, 0.1% (v:v) Tween 20. Thereafter, coverslips were incubated overnight with BG4 single chain antibody (1 ng/µL) in a humid chamber at 4 °C. After washing with PBS 1x, sensitive detection was achieved through an amplified fluorescence signal generated by incubation

for 1 h at room temperature with anti-FLAG antibody produced in rabbit (Sigma cat. F7425) at 5 ng/µL. Thereafter, anti-rabbit Alexa Fluor™ 488 (5 ng/µL, ThermoFisher Scientific) as a tertiary fluorochrome-labelled antibody (1 h at RT). Coverslips were mounted into glass slides using mounting media containing DAPI (5 ng/µL). Images were acquired with a confocal microscope (Zeiss LSM 980) using a × 63 magnification objective (Nikon). Telomere spots were quantified with Fiji software using the Find Maxima tool with variable values of prominence depending on each experiment: each cell was analysed, and quantification (number of *foci* identified) made by the software was also checked and confirmed by eye-inspection. Co-localisation rate of telomere spots with BG4 *foci* were quantified by eye-inspection considering the sum of green (BG4 *foci*) and red (TelG-Cy3) resulting in a yellow signal (co-localisation) as G4-forming telomeres.

FANCJ protein expression and purification - The human FANCJ DNA helicase was produced as described in *Materials and Methods* section of the main text.

Gel-based helicase activity assay - DNA oligonucleotides were purchased from Biomers (Ulm, Germany) and listed in Table S2. G4 DNA structures were formed by heating mixtures containing each indicated oligonucleotide at a concentration of 1 µM in annealing buffer (10 mM Tris-HCl, 1 mM EDTA, 100 mM KCl, pH 7.5) for 2 min at 90 °C. Then, each mixture was subjected to slow cooling (90 s/1 °C) up to 10 °C to allow annealing. FANCJ (40 nM) was incubated in reaction mixtures (volume: 20 µL) containing each indicated FAM-labelled G4 DNA substrate (10 nM) in Buffer H (25 mM HEPES-NaOH, pH 7.2, 100 mM KCl, 5 mM $MgCl_2$, 2 mM DTT, 0.01 % [w:v] BSA) for 2 min at room temperature to allow the protein–DNA complex to form. Then, increasing concentrations of FIM-15 compound were added to the reaction mixtures. G4 resolution was initiated by adding ATP (2 mM) together with an excess of a capture DNA oligonucleotide (200 nM) that hybridised with the sequence forming the G4 structure, thereby preventing it from reforming. After an incubation for 15 min at 30 °C, the reactions were quenched with the addition of 5 µL of 5 × Stop Solution (0.5 % [w:v] SDS, 40 mM EDTA, 0.5 mg/mL [w:v] proteinase K, 20 % [v:v] glycerol). Samples were incubated for 5 min at 20 °C and then, run on a 12.5 % polyacrylamide-bis (19:1) gel in TBE 1x containing 100 mM KCl at a constant voltage of 90 V on ice. The individual bands were visualised on a ChemiDoc MP imager, and the band intensities were determined using Image Lab software (Bio-Rad Laboratories).

Table S1 – IC_{50} values of the indicated G4-stabilisers

	IC_{50} [µM]		95 % Confidence interval [µM]	
	CTRL	*FANCJ*$^{-/-}$	CTRL	*FANCJ*$^{-/-}$
PDS	1.644	1.118	1.392 to 1.941	0.9224 to 1.356
FIM-20	6.519	7.705	5.511 to 7. 712	6.343 to 9.361
CBR-15	0.6893	0.5807	0.4953 to 0.9593	0.4395 to 0.7672
FIM	0.7626	0.7588	0.6273 to 0.9269	0.6307 to 0.9129
FG	2.758	1.8	2.366 to 3.215	1.502 to 2.157
FIM-15	0.8798	0.4357	0.6908 to 1.121	0.3586 to 0.5293

Table S2 – Sequences of the DNA oligonucleotides used for helicase activity assay

Name	Sequence
c-Myc	5’-AAAAAAAAAAAAAAAAAAAAATGAGGGTGGGTAGGGTGGGTAA-3’
c-Kit1	5’-AAAAAAAAAAAAAAAAAAAAAAGGGAGGGCGCTGGGAGGAGGG-3’
Tel_{26}	5’-AAAAAAAAAAAAAAAAAAAAATTAGGGTTAGGGTTAGGGTTAGGGTT-3’
c-*Myc* capture	5’-ACCCTACCCACCC-3’
c-*Kit*1 capture	5’-TCCTCCCAGCGCCC-3’
Tel_{26} capture	5’-CCCTAACCCTAA-3’

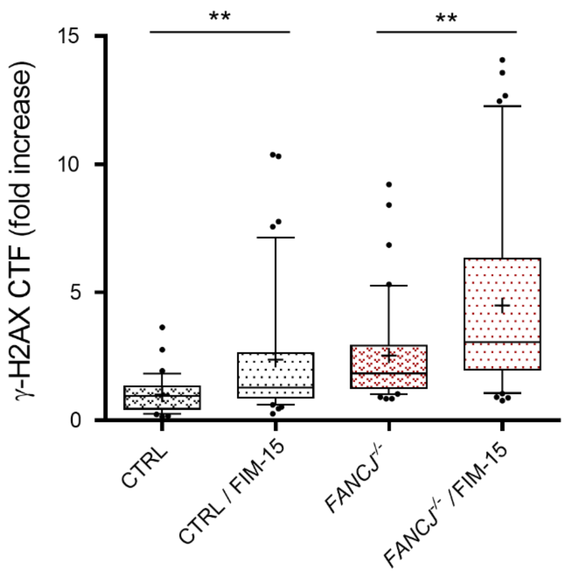

Figure S1. Quantitative analysis of DNA damage upon FIM-15 treatment. Fold increase of γ-H2AX corrected total fluorescence (CTF) per nucleus in the indicated conditions. Treatment was performed for 24 h with FIM-15 (250 nM). The chart reports medians (lines), mean (cross), 50 % (box) and 90 % (whiskers) of the data sets and outliers (circles). A total of 100 cells were analysed per condition in three biological independent experiments. Statistical analyses were performed using a non-parametrical Mann-Whitney U test. Calculated p values were: **, $p < 0.01$.

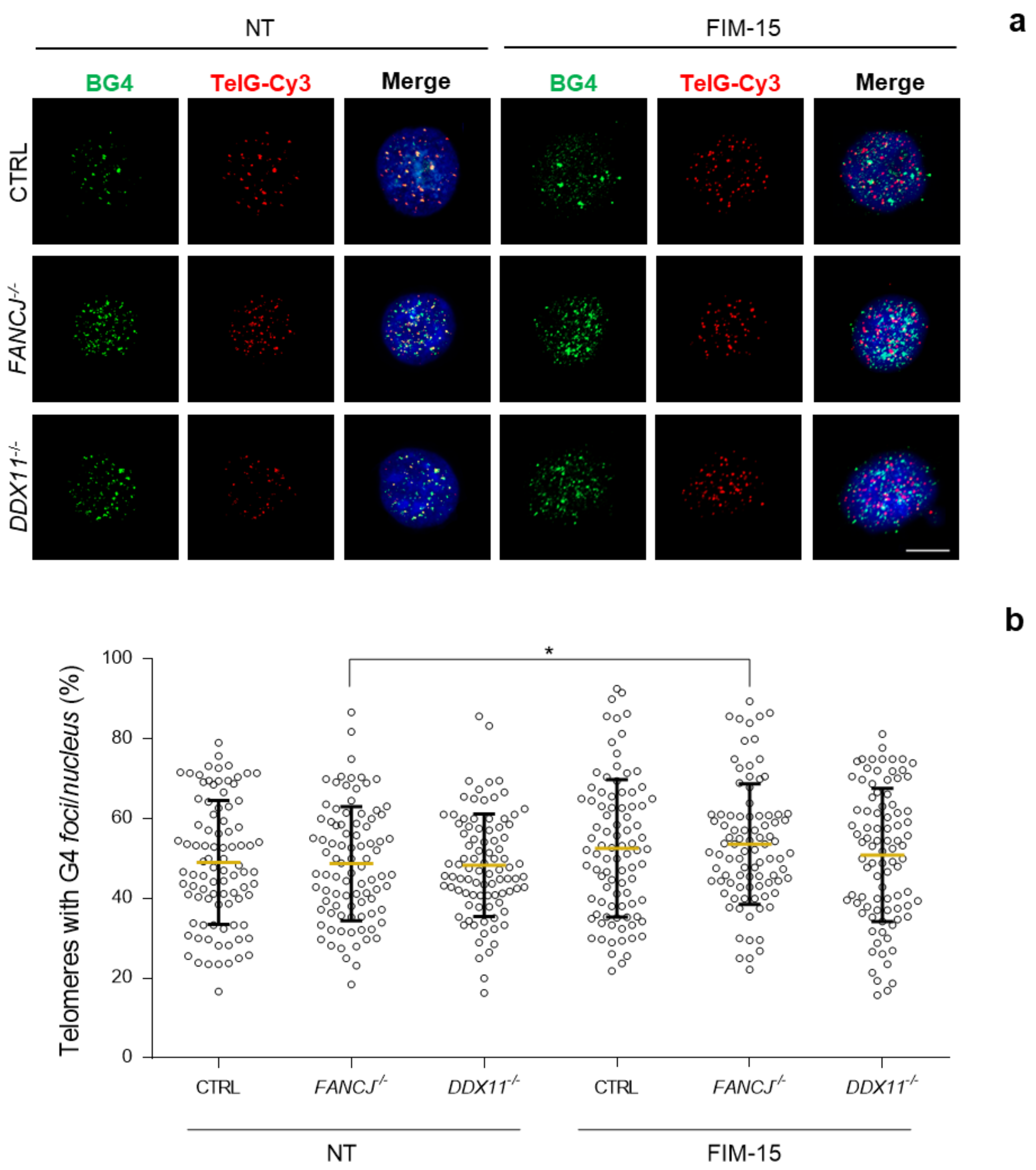

Figure S2. Occurrence of G4 structures at telomeres in FIM-15-treated *FANCJ*- and *DDX11*-KO cells. (a) Representative images of BG4 *foci* (IF-green) co-localising with telomeres (FISH-red) that result in a yellow signal in interphase nuclei of the indicated cell lines. Scale bar, 5 µm. (b) Percentage of G4-containing telomeres per nucleus in the indicated cell lines. The chart reports mean (yellow line) ± SD. Each spot represents a single nucleus (90 nuclei were analysed for each condition in 3 biological replicates). Statistical analyses were performed using a non-parametrical Mann-Whitney U test. The reported p value was *, $p < 0.05$.

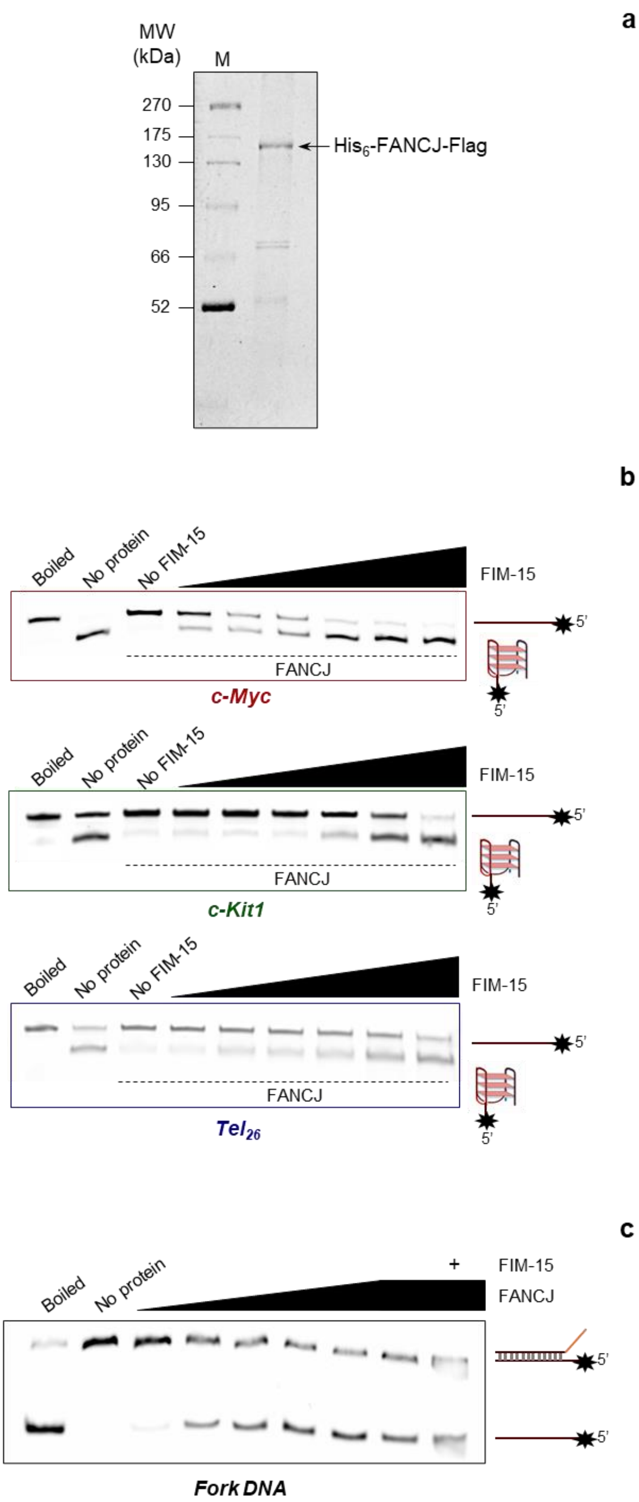

Figure S3. FANCJ gel-based DNA helicase assays. (a) SDS-PAGE analysis of human His6-FANCJ-Flag, purified from transiently transfected HEK 293T cells. M indicates the lane containing a protein marker. (b) FANCJ helicase activity assays were carried out using 40 nM of protein and increasing concentrations (0.25, 0.5, 0.75, 1 and 10 μM) of the FIM-15 compound with the indicated fluorescent-labelled unimolecular G4 DNA substrates. Lane, named "Boiled" in each gel, contains a heat-denatured assay mixture with no protein. Lane, named "No protein" in each gel, contains a mock assay without FANCJ. Lane, named "No FIM-15" in each gel, refers to an assay carried out in the absence of the compound. (c) FANCJ helicase activity assays were carried out using increasing concentrations of the recombinant protein (5, 10, 20, 40, 80 and 160 nM) with a fluorescent-labelled double stranded DNA as a substrate. Lane, named "Boiled", contains a heat-denatured assay mixture with no protein. Lane, named "No protein", contains a mock assay without FANCJ. Lane, named "+", refers to an assay carried out in the presence of FIM-15 (1 μM) and the highest concentration of FANCJ protein (160 nM).

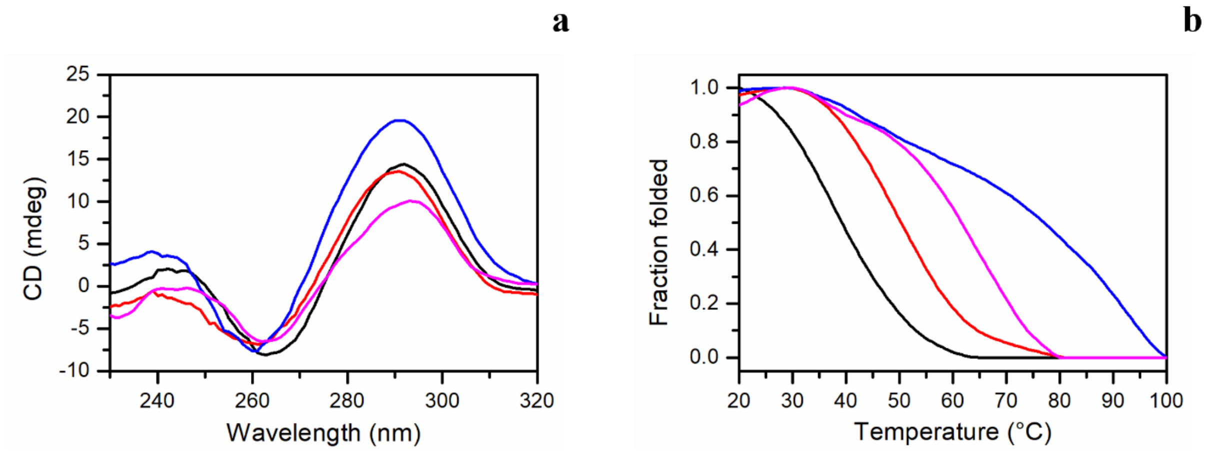

Figure S4. Biophysical characterization of the G4 DNA structure formed by the Ce20 oligonucleotide. (a) CD spectra of Ce20 (black line) with 2 mol equiv. of FIM-15 (red line), PhenDC3 (blue line) and PDS (magenta line). (b) CD melting experiments of Ce20 G4 (black line) with 2 mol equiv. of FIM-15 (red line), PhenDC3 (blue line) and PDS (magenta Line).
